# A framework for understanding outcomes of integrated care programs for the hospitalised elderly

**DOI:** 10.5334/ijic.1063

**Published:** 2013-11-28

**Authors:** Jacqueline M. Hartgerink, Jane M. Cramm, Jeroen D.H. van Wijngaarden, Ton J.E.M. Bakker, Johan P. Mackenbach, Anna P. Nieboer

**Affiliations:** Department of Social Medical Sciences, Institute of Health Policy and Management, Erasmus University Rotterdam, Rotterdam, The Netherlands; Department of Social Medical Sciences, Institute of Health Policy and Management, Erasmus University Rotterdam, Rotterdam, The Netherlands; Department of Health Service and Management of Organizations, Institute of Health Policy and Management, Erasmus University Rotterdam, Rotterdam, The Netherlands; Argos Zorggroep, Voorberghlaan, Schiedam, The Netherlands; Department of Public Health, Erasmus Medical Center Rotterdam, Rotterdam, The Netherlands; Department of Social Medical Sciences, Institute of Health Policy and Management, Erasmus University Rotterdam, Rotterdam, The Netherlands

**Keywords:** Framework, integrated care, elderly, hospital, team work, professional collaboration

## Abstract

**Introduction:**

Integrated care has emerged as a new strategy to enhance the quality of care for hospitalised elderly. Current models do not provide insight into the mechanisms underlying integrated care delivery. Therefore, we developed a framework to identify the underlying mechanisms of integrated care delivery. We should understand how they operate and interact, so that integrated care programmes can enhance the quality of care and eventually patient outcomes.

**Theory and methods:**

Interprofessional collaboration among professionals is considered to be critical in integrated care delivery due to many interdependent work requirements. A review of integrated care components brings to light a distinction between the cognitive and behavioural components of interprofessional collaboration.

**Results:**

Effective integrated care programmes combine the interacting components of care delivery. These components affect professionals’ cognitions and behaviour, which in turn affect quality of care. Insight is gained into how these components alter the way care is delivered through mechanisms such as combining individual knowledge and actively seeking new information.

**Conclusion:**

We expect that insight into the cognitive and behavioural mechanisms will contribute to the understanding of integrated care programmes. The framework can be used to identify the underlying mechanisms of integrated care responsible for producing favourable outcomes, allowing comparisons across programmes.

## Introduction

Population ageing presents a great challenge to our society. Since the incidence of chronic disease increases with age [1], the number of elderly requiring hospitalisation for chronic diseases is expected to increase proportionally [2]. Once admitted to the hospital, older adults are at an increased risk of poor outcomes such as readmission, increased length of stay, functional decline, iatrogenic complications and nursing home placement [3,4]. Given that 34–50% of hospitalised older adults have been found to experience functional decline [5,6], it is likely that traditional health care delivery does not meet the needs of an ageing population. The complex needs of older patients ask for the coordination of health and social care with related services delivered by multiple providers [7]. Hospital care that does not address the functional needs, psychosocial issues and altered response of older patients to illness and treatment [8,9], puts older patients at risk of receiving fragmented or poor-quality care [10,11]. Recognition of the shortcoming has led to the new strategies of care delivery such as integrated care programmes [12], which are expected to resolve many problems surrounding elderly care.

Quality improvement programmes in hospitals usually focus on isolated interventions, such as medication supply or multidisciplinary cooperation, rather than integrated programmes that incorporate the total care process [13]. The World Health Organization [10] defined integrated care as a holistic and personalised approach to multidimensional health needs. It reduces the duplication and fragmentation of care while improving coordination and continuity by placing the elderly central to the health care delivery process [7,14]. With the integration of interrelated care delivery components (e.g. case management, support systems, multidisciplinary teamwork, treatment plans), the system is reformed such that informed patients and their caregivers can interact with proactive professional teams. Such a reform positively affects the quality of care and patient outcomes [7,15,16]. De Morton and colleagues [17] found that multidisciplinary interventions resulted in an increased proportion of discharged patients, shorter hospital stays for elderly patients with acute conditions and lower hospital costs. No similar effect was found with implementing a single intervention. In addition, Caplan and colleagues [18] found that a comprehensive geriatric assessment followed by implementation of integrated care with multidisciplinary team interventions improved the health outcomes of elderly patients at risk of physical deterioration during hospitalisation [19,20]. The hospital treatment of vulnerable elderly patients currently focuses on diagnostics; it should also focus on the integration of health and social care with related services in a multidisciplinary context [7].

Little is known about the underlying mechanisms that explain the effectiveness of integrated care for elderly patients. Theories of integrated care have two opposing strategies: gradual and radical redesign in the steps of providing integrated care, by comprehensive, organisation-wide efforts to improve quality [21]. Regardless of the strategy, however, the complexity and multidisciplinary settings of integrated care programmes pose difficulties for implementation. Like other quality improvement interventions, they are complicated by the variety of approaches among organisations and multidisciplinary teams within the same organisation [22]. Each professional's individual care delivery and adherence to clinical guidelines adds more variation.

Integrated care must be organised such that the services intended to produce the desired outcome can and will be provided [7]. Too often, interventions are evaluated without first gathering data that describe the processes mediating improvements [22]. Current measures of quality in health care, such as the structure-process-outcome model, do not clarify the underlying mechanisms governing the components of integrated care [23–25]. The relationship between structure and outcome often remains unclear because a sound analytical method for evaluating the outcomes of integrated care programmes, which would provide insight into why and where they are effective, is lacking. In this article, we provide a framework to increase our understanding of the relation between structure and outcomes by explaining the underlying mechanisms of the process of integrated care delivery to elderly provided by professionals within hospitals. The overall aim of this framework is to identify the underlying mechanisms of integrated care delivery and to understand how they operate and interact, so that integrated care programmes can enhance quality of care delivery and eventually patient outcomes.

## Pillars of integrated care delivery

Interprofessional collaboration among professionals from a variety of disciplines is considered to be critical in integrated care delivery due to the many interdependent work requirements [26–29]. To provide integrated care that is holistic and patient-centred responding to the multidimensional health needs of older patients more is needed than professionals who each work within their particular scope of practice and interact formally (multidisciplinary teamwork), but rather professionals who have some overlapping of professional roles, communicate and coordinate together in their care of older patients and share problem solving and decision making (interprofessional collaboration) [30–32]. In this way, the coordinated response of all activities and information to the needs of older patients is organised through horizontal work processes, rather than through functional profiles, creating interdisciplinary instead of multidisciplinary care delivery.

The literature provides some indications of what interprofessional collaboration entails; yet, it demonstrates that we have limited understanding of the complexity of relationships between professionals and underlying mechanisms of the delivery of care to elderly provided by professionals in a multidisciplinary context [33–35]. A review of the delivery of integrated care components by professionals brings to light a key distinction between the cognitive and behavioural components of interprofessional collaboration. Effective integrated care programmes often involve new professional collaborations, task reallocation, communication improvements, case management and the use of new types of professionals [7,15]. Such changes in care provision affect the cognition and behaviour of professionals, which in turn affect the quality of their delivery of care to patients [36,37]. We thus expect that cognitive and behavioural perspectives on the delivery of integrated care through interprofessional collaboration will contribute to our understanding of the effects of such changes. While both perspectives share the same objective, their processes vary. The cognitive perspective explains changes in care delivery in terms of the mental states of professionals [38,39]; the behavioural perspective holds that changes in care delivery result from interaction among professionals [40,41]. The closely related situated cognition and behaviour influence each other, just as cognition and behaviour are influenced by team [42] and organisational [27,43–45] contexts.

## Development of the framework

**Figure 1.**
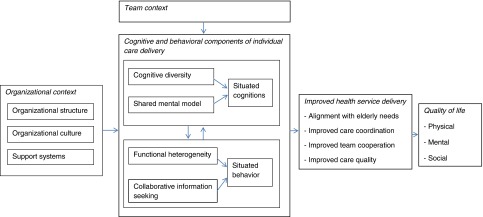
Evaluation model for integrated care programmes for elderly in hospitals.

Our framework is based on the principles of programme theory, which consists of a set of statements that describe a particular programme, explain why, how and under what conditions the programme effects occur, predicts the outcome of the programme and specifies what needs to be done to bring about the desired outcomes [46,47]. A review of literature on integrated care programmes [12,48–50] allowed us to define the presenting problem and to unravel the changes in care delivery that are expected and the way in which change is to be achieved. The theory of organisational knowledge creation helps to understand the process of making knowledge available and amplifying knowledge created by individuals as well as connecting it to others’ knowledge [51]. This theory allowed us to identify the cognitive and behavioural mechanisms underlying interprofessional collaboration and integrated care delivery. By identifying the underlying mechanisms of integrated care delivery we may increase our understanding of how they operate and interact in order to enhance quality of care and eventually patient outcomes. The main purpose of the evaluation model is to test the programme theory, and to identify what it is about the programme that causes the intended outcomes. The evaluation model incorporates variables that reflect theoretical concepts of integrated care evaluation for elderly in hospital settings. In the following sections, we conceptualise integrated care processes to identify the mechanisms responsible for producing intended outcomes. Such a theoretical structure selects outcomes that correspond to improved service delivery (aligning care with the needs of elderly patients, coordination and collaboration, resource utilisation [12]) and reflect the primary goal of integrated care, namely, enhancing patients’ quality of life (Figure 1).

First, the cognitive components of the framework are outlined. These components consist of mechanisms that alter the way of thinking by professionals delivering care to older patients. Next the behavioural components are explained, which consist of mechanisms that explain how professionals actively share and combine patient information from various sources. Since professionals do not work in isolation but operate in teams, they are affected by the team in which they work. Teams working in cardiology, for example, may work differently together compared to teams in the geriatric or orthopaedic departments. Furthermore, team functioning is expected to be influenced by the organisations in which they work. Research indeed demonstrated that team functioning is affected by organisational characteristics [52–54].

### Cognitive components of individual care delivery

Cognitive components of individual care delivery are cognitive diversity and a shared mental model, which together result in situated cognitions. Examining situated cognition awareness of team members leads to an important perspective for a system design that supports teams’ complex interrelated activities. Endsley [55] has defined team-situated cognition awareness as the degree to which every team member possesses the situated cognition awareness required for his or her responsibilities. Effective integrated care requires that each team member possess the knowledge required for optimal patient care and interventions designed to integrate the discrete areas of expertise [15]. Cognitive diversity reflects the differences in team members’ knowledge, beliefs, preferences and perspectives [56]. The integration of these diverse cognitions within interdisciplinary teams, reflecting the knowledge and skills of different disciplines, increases the likelihood of new knowledge development [56,57]. An integrated approach is particularly suitable to complex health problems, such as co-morbid and frail elderly hospital patients. Ideally, each interdisciplinary team member knows the diverse points of view held by all other professionals on the team, and trusts them to deliver the necessary care. Critical elements of care are expected to be completely delivered by combining the existing cognitive capacities and capabilities of each member [58].

Situated cognition awareness is also influenced through shared mental models of interdisciplinary team members. Researchers have provided substantial evidence that mental models have strong effects on perceptual processes and organisational outcomes [59–61]. Endsley and Jones [62] define shared situated cognition awareness as ‘the degree to which team members possess a shared understanding of the situation with regard to their shared situated cognition awareness requirements’. Measureable shared cognitive perceptions of organisational policies, practices and procedures can be manipulated to enhance the effectiveness of a team [42,63]. Shared objectives, commitment and support positively relate to the continuous delivery of high-quality care [42,64]. This connection is primarily based on the understanding that, through the integration of diverse knowledge, teams have the potential to overcome the factors constraining the development of new knowledge by social relations [57]. By extended interaction and shared practice team members have identical experiences and, eventually, comparable interpretations of those experiences, with the added value of team members knowing ‘who knows what’ [57,65]. The development of such a shared mental model enables team members to form the same psychological representations, resulting in accurate explanations and expectations of others’ actions [66]. Interdisciplinary team members who share the same mental model are expected to excel at aligning care with the needs of elderly patients as they are better able to provide a thorough problem description and needs assessment, and to develop common treatment goals and standards that coordinate care delivery. We hypothesise that the added value of cognitive diversity and the combined value of a shared mental model improve interprofessional collaboration and lead to more effective integrated care delivery.

The integration of cognitive diversity and a shared mental model leads to the situated cognition awareness required to deliver high-quality integrated care. This arises from interactions of existing cognitive structures within the multidisciplinary team context [37]. Because the hospital context is both social and dynamic, situated cognitions tend to be transitory, formed by the interaction of existing cognitive structures and treatment of a specific patient. The cognitions of an individual professional adapt to specific contexts of individual patients, making it possible to align care to the specific needs of each elderly patient. We hypothesise that the effects of integrated care delivery result from situated cognitions, which originate with cognitive diversity and shared mental models of health care professionals’ interaction with patients.

### Behavioural components of individual care delivery

Effective integrated care requires optimal professional behaviour. Behavioural components of individual care delivery – functional heterogeneity and collaborative information-seeking – combine to result in the development of situated behaviour.

The integration of the behaviour of diverse professionals within a team is central to the success of integrated care [67]. Interdisciplinary teams, which embody heterogeneous roles, express more diverse opinions about the tasks, procedures and appropriate actions than homogeneous teams [68,69]. Functional heterogeneity defines teams that are diverse in terms of the occupational background and encourages individuals to adapt their behaviour to that of other disciplines represented by the team, in order to provide a complete overview of the elderly situation. Professionals provide each other with feedback regarding appropriate care. As a result, multidisciplinary treatment plans are expected to form a coherent whole in which individual professionals’ actions are combined and new types of professionals can be introduced. In doing so, interdisciplinary teams help elderly patients navigate the complexities of multiple health problems, while receiving less fragmented and duplicated care [58].

Multidisciplinary team meetings constitute the basic prerequisite for collaboration [70,71]. Professionals are thereby involved in collaborative information-seeking to address a specific problem, and use each other as information sources [70,72], facilitating the coordination of appropriate actions in the delivery of care to elderly patients. Clinical information from different disciplines is transferred more effectively, enhancing the early detection of health problems and the adaptation of care delivery actions. Functional heterogeneity and collaborative information-seeking are both expected to be underlying mechanisms leading to effective integrated care programmes. Social and behavioural theories are useful for gaining an understanding of the influence of behavioural processes within multidisciplinary teams. Activity theory, for example, states that the division of labor in an activity creates a distinct position for each team member. Members bring diverse histories to the team; the activity system contains multiple layers. This multi-vocality is multiplied in networks of interacting activity systems, which may lead to collaborative envisioning and a deliberate effort to bring about collective change [73]. Clinical information-seeking practices have been shown to be distributed throughout the professional team through the process of collectively seeking, interpreting and assessing information, especially in the case of multiple care components [74,75]. We hypothesise that the added value of functional heterogeneity and collaborative information-seeking will improve interprofessional collaboration and lead to more effective integrated care delivery.

An adequate behavioural response emerges as the interaction of individual and team behaviour with the environment. A common goal of an integrated care intervention is to increase the knowledge and expertise necessary for the care of elderly patients; the behaviour of professionals must be adapted to each medical situation. Such situated behaviour emerges when the mechanisms of functional heterogeneity and collaborative information-seeking are aligned with the needs of elderly patients. Professionals’ behavioural intentions will thereby be affected, which we expect to lead to improved health service delivery and enhanced quality of life.

### Team context

The cognitions and behaviour of professionals are influenced by team context: its history, duration, performance record, resources, leader stability, member abilities, size and level of diversity [42]. Interdisciplinary teams – diverse in terms of occupation and function – are likely to be diverse in other ways as well. Evidence for the influence of team context mechanisms supports the reasoning that care delivery benefits from teamwork. A team context, however, must support teamwork in a way that integrates cognition and behaviour into a coherent system.

### Organisational context

Supportive organisational systems, structure and culture are known to promote effective integrated care delivery for older patients [43–45]. Activities like integrated care can only grow out of individuals interacting with the organisational context [36]. Organisational theorists define structure as the configuration of relationships with respect to responsibilities, authority and task allocation [44,76]. Health care organisations are typically characterised by centralised authority, work regulation and formalisation [77]. Such structuring consists of separate chains of control for different professionals [78]. In contrast, integrated care delivery requires a more organic mode of structuring that incorporates flexible working processes, enabling the introduction of new instrumental and technical working methods. Effectiveness arises from identification with the new professional role within the interdisciplinary team [79]. An organisational structure in which flexible task structuring and information sharing are facilitated should thus yield to the integration of professional cognition and behaviour, thereby increasing the quality of care delivery.

Organisational culture is expressed by the pattern of shared assumptions – invented, discovered or developed by an organisation as it learns to cope with problems – that are taught to new members as the correct way to act [43,80–82]. Organisations benefit most from integrated care programmes when teamwork, coordination and customer focus are emphasised. Formal structuring and regulations appear to be negatively associated with quality improvement activities [83–85]. Professionals working in interdisciplinary teams face the relatively new and unfamiliar position of defending their established professional work domains, in contrast to the well-defined hierarchical power structure of traditional, physician-controlled cultures [86–88]. The diversity among interdisciplinary team members challenges the established hierarchy's power and authority.

Support systems (e.g. information, communication and clinical guidelines) improve the planning of care delivery based on clinical investigations and outcomes [89]. An integrated care programme often involves the construction of new support systems that change the instrumental and technical aspects of care delivery. They allow patients and professionals to be properly informed and can improve quality of care by facilitating the provision of feedback to professionals on outcomes [45,90]. Timely information about patients has been proven to be a common feature of effective care [58]. The most basic need is to establish a registry that includes information on the performance of various aspects of guideline-informed care. Interdisciplinary teams with access to such a registry can deliver proactive care, receive feedback, implement reminder systems, generate tailored treatment plans and provide patient- or provider-specific messages to facilitate integrated care delivery [91]. Information and communication systems facilitate the integration of services to improve overall performance [92].

### Improved health service delivery and quality of life

Research shows that integrated care results in improved delivery of care aligned to individual needs of patients [7,15,16]. Collective learning theories emphasise that mental models, such as cognitive diversity and shared mental models, are used as a basis for modifying and optimising the mechanisms underlying the effectiveness of integrated care delivery [93,94]. Although much remains to be learned about the influence of such cognitive models in a hospital setting, some empirical evidence is currently available. Medical facilities that excel in providing diagnostic and procedural information have been shown to exhibit a shared mental model through similar conceptions of guidelines [95]. This finding is consistent with previous research using cognitive models, which has linked cognitive diversity and shared mental models to improved team cooperation and coordination [96,97]. Improved cooperation and coordination result in more effective evaluation and planning of elderly patients’ needs. Our framework illustrates that professional situated cognitions and behaviours position the complex holistic needs of the elderly central to health service delivery. The added value of the cognitive and behavioural components for interprofessional collaboration is expected to result in enhanced coordination and cooperation, better quality of care and alignment with elderly patients’ needs [7,15,16,98]. The interrelated components of team cooperation, care coordination, quality of care and alignment with elderly patients’ needs are integrated through situated cognition and behaviour. We hypothesised that the integration of holistic and personalised health care improves a patient's quality of life. Integrated care is known to provide these opportunities [12,17,18,99–101].

### Example of an application of the evaluation model: evaluating an integrated care programme for the hospitalised and vulnerable elderly

**Figure 2.**
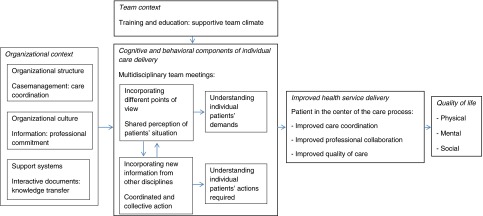
Example of an application of the evaluation model on an integrated care programme for the hospitalised and vulnerable elderly.

About 35% of elderly patients admitted to hospitals function less well after discharge than prior to admission [19]. The loss of function is associated with the disorder for which the patient was admitted, but the hospital stay itself also leads to health problems. The Prevention and Reactivation Care Program, an integrated care programme, was designed to prevent loss of function in elderly patients due to hospitalisation, targeting patients 65 years or older who are vulnerable to function loss after discharge. The programme supports a multifaceted and multidisciplinary approach to elderly care. The care is organised around several core components, including screening for vulnerability, early detection of health problems, multidisciplinary teamwork and case management [102]. The framework provides a valuable starting point for understanding the underlying mechanisms of the Prevention and Reactivation Care Program responsible for producing a favourable outcome (Figure 2).

#### Organisational context

- *Structure:* Within 48 hours of admission, the level of vulnerability of the elderly patient is determined with a screening instrument. Through Goal Attainment Scaling, an individual treatment plan is formulated. A case manager, who places the elderly and their caregivers central to the health care delivery process, is assigned to the patient throughout the integrated care spectrum, from hospital to nursing and home care. The organisational structure is characterised by the case manager's representation of the elderly within interdisciplinary teams and frequent patient interaction, which facilitates information sharing and provides input for the development of professional cognitions and behaviour. In this way, the case manager serves as a care coordinator between professionals, as well as between patients and professionals.
- *Culture:* Information booklets describing the programme and corresponding protocols are distributed among hospital professionals. Formal arrangements of face-to-face discussion stimulate professionals to share new ideas and insights and keep professionals up-to-date about developments [103]. The periodic presentation of programme results is expected to create a stimulating learning and supportive environment for programme implementation. In this way, professional commitment is achieved.
- *Support systems:* Support systems are designed to enhance information transfer among professionals. Available interactive documents include individualised support for professional practices and several practical implementation tools (patient assessment, Goal Attainment Scaling scores, individual treatment plans, chart stickers/vital sign stamps and advice scripting). The use and content of this information system provides an indication of programme implementation.

#### Individual care delivery by interdisciplinary teams

- *Cognitive component:* Effective integrated care requires every team member to possess the situated cognition awareness required for his or her responsibilities. This dynamic understanding of ‘what's going on’ makes it possible to organise care around patient needs. The functional diagnosis in relation to the medical diagnosis of the elderly is discussed by the geriatrician, nursing home physician, social worker and case manager. An individual treatment plan that emphasises patients’ functional status is formulated by incorporating team members’ different points of view. Multidisciplinary team meetings enhance the formation of common goals and treatment standards. Instead of incorporating only their own viewpoints of the patients’ situation, this generates a shared mental model of the patient's situation. The greater the degree to which team members possess this mental model, the better their ability to interpret information similarly and make accurate projections regarding each other's action. Using clinical guidelines and protocols for integrated care treatment promotes shared cognitive perceptions, practices, objectives and procedures. As a result, their shared perception of the actual situation of the patient (e.g. awareness of the current health condition), in combination with a comprehension of what might be necessary for the patient (e.g. knowledge about different treatment options), and a projection of what might happen (e.g. how to react to sudden deterioration) allows professionals to better respond to each patient's personal needs [104–106].
- *Behavioural component:* Multidisciplinary team meetings enhance information sharing. Emphasis on the value of professional feedback and individual input during these multidisciplinary team meetings, which can be scored directly, increases effective interprofessional collaboration. Professionals who are provided with the opportunity to connect with other professionals through formal activities are expected to expand their professional knowledge and skills [107]. In addition, it is important that professionals also timely inform others when new patient information emerges [108]. These formal communication methods and the relational dynamics provide the basis for coordinated collective action that places the elderly in the centre of the care process [109,110].
  As a result, professional actions are combined through sharing diagnostic and clinical patient information, and the number of consulted professionals is likely to increase. In this way, care delivery is less fragmented and duplicated. Individual health problems are detected early, avoiding complications and future health problems. Case management enhances such processes by gathering information about the patient from different professionals. Combining these diverse information sources is a criterion for adapting professional actions to the elderly patient's needs.

#### Team context

- A case manager is assigned to each elderly patient and is responsible for this patient in the total process of care, which promotes leader stability. Team member performance, resources and abilities improve through specialised training and education. This results in a supportive climate for teamwork, as team members are more willing to share resources, perceptions, policies, practices and procedures [80]. As such, a team climate may encourage social interaction and draws the interpretations by professionals of events and objects closer together [111,112].

#### Health service delivery

- Cognitive diversity and shared mental models facilitate coordination and cooperation between interdisciplinary team members, by creating a dynamic understanding of the individual patients’ situation. Situated cognition awareness and behaviour results in team members’ checking each other for conflicting information or perceptions. Tasks are coordinated and prioritised, and contingency planning is established, placing the patient in the centre of the care process.
- Shared situated cognitions and behaviour leads to timely, accurate, demand-driven care aligned with needs of the elderly patient. Intermediate outcomes are thus also defined to reflect changes in health service delivery resulting from the improvements. Improved coordination in care delivery and professional cooperation decreases duplication and fragmentation of care.

#### Quality of life for elderly patients

The integration of diverse cognitions and shared mental models within interdisciplinary teams is translated into multidisciplinary treatment plans that describe the care needed by an individual patient. Integrated care delivery has shown to improve the quality of care due to patient involvement in planning of care, better patient education, more staff time with patients and improved communication between professionals [113]. In this way, the perceived quality of integrated care by the patient affects patient outcomes (Hartgerink et al., unpublished observations). Providing care with a holistic and personalised integrated care approach prevents the loss of function in elderly patients due to hospitalisation, aligns care with their needs, and enhances their quality of life.

## Discussion and conclusion

Integrated care programmes in hospitals are assumed to be a systematic and comprehensive approach to improve the management of complex health problems. Evidence for these improvements is currently lacking and a sound analytical method for evaluating the outcomes of integrated care programmes has to this date been unavailable. Our framework provides a valuable starting point for doing so. The theory of organisational knowledge creation is used to explain how knowledge is made available, how it is amplified and how knowledge from different professionals is connected [51]. Because interprofessional collaboration is the core component influencing the effectiveness of integrated care delivery, professionals’ cognitions and behaviours are of primary importance. Programme evaluation should thus focus on the combined effects of these behavioural and cognitive components in relation to the specific needs of elderly patients. Programme theory shows that team and organisational support are also indispensable. The use of supplementary interventions on these levels strengthens the effect of the programmes.

Most models and theories are based on the same principles for the successful implementation of changes in health care delivery: a systematic and sequential approach, commitment of the relevant population, process monitoring and implementation planning [84,114–119]. We know that successful interventions provide the patient with case management, professional feedback, explicit protocols, support systems and reorganisation to better meet patient needs within a multidisciplinary context [7,15,16,120,121]. Yet, there exists no explanatory theory of the mechanisms by which integrated interdisciplinary teamwork affects care outcomes. Lemmens and colleagues [122] tried to open this black box by conceptualising the change mechanisms of the patient and professionals. But they did not make explicit the professionals’ cognitions and behaviours in interprofessional collaboration, or the combined effects of these components in relation to the specific needs of elderly patients. Our evaluation framework elucidates the mechanisms underlying the working components of integrated care interventions within an elderly care setting. Situated cognitions focus on the process of thinking and acting by individual professionals. A dynamic understanding of patients’ specific situation and needs places the elderly person in the centre of the care process, making it possible to consider and balance different treatments to each patient. Situated behaviour assumes a continuous interaction among the professional, his or her performance, and the elderly, which reinforce one another in changing behaviour. The actions professionals take while delivering care and the way diverse information is combined enables a focus on how professionals coordinate their tasks in ways that meet the individual patients’ demand for care. The identification of these mechanisms, rooted in cognitive and organisational psychology, facilitated the construction of the evaluation framework. What this framework adds to current models and theories is a deeper understanding not only of the integrated care interventions but also of the underlying mechanisms responsible for producing a favourable outcome. Besides understanding these underlying mechanisms, it is of importance for the evaluation of integrated care programmes, to understand the degree to which suggested integrated interventions are actually performed. A fidelity study can be used to determine the extent to which the intervention was delivered as intended [123].

In testing the relations presented in the framework, we found empirical evidence that the behavioural components of professionals delivering care to older patients are indeed of importance for integrated care delivery [109]. In addition, we found that, consistent with our evaluative framework, the cognitive components of professionals delivering care to older patients are associated with integrated care delivery (Hartgerink et al., unpublished observations). Furthermore, team context and organisational context affect interprofessional collaboration and integrated care delivery [110] (Hartgerink et al., unpublished observations). These are important findings and support our expectations that the underlying behavioural and cognitive mechanisms are important for integrated care delivery to older patients.

## Implications of findings

Earlier research discussed the importance of providing insight into why interventions for older patients are effective [24,124]. The current framework suggests that the underlying cognitive and behavioural components, and team and organisational context are important for the effectiveness of integrated care programmes in hospitals. The theoretically derived relations should be tested in empirical research. Programmes can then be analysed by deconstructing the framework's components, allowing cross-comparison of different programmes. Consistent use of the framework will therefore enable valid comparison of the outcomes of integrated care programmes. To do so, integrated care programmes should be evaluated systematically, by for example, developing integrated care specific indicators [125].

In an era plagued by ever-tightening health care resources and characterised by an ageing population, it is of utmost importance to identify interventions that add value to the quality, efficiency and effectiveness of care for vulnerable elderly patients and understand the underlying mechanisms of why interventions work. The current framework provides an essential first step, by examining how integrated care delivery affects processes and outcomes of care.
